# The Auditory Brain-Stem Response to Complex Sounds: A Potential Biomarker for Guiding Treatment of Psychosis

**DOI:** 10.3389/fpsyt.2014.00142

**Published:** 2014-10-13

**Authors:** Melissa A. Tarasenko, Neal R. Swerdlow, Scott Makeig, David L. Braff, Gregory A. Light

**Affiliations:** ^1^VISN-22 Mental Illness, Research, Education and Clinical Center (MIRECC), VA San Diego Healthcare System, La Jolla, CA, USA; ^2^Department of Psychiatry, University of California San Diego, La Jolla, CA, USA; ^3^Swartz Center for Computational Neuroscience, Institute for Neural Computation, University of California San Diego, La Jolla, CA, USA

**Keywords:** auditory brain-stem response, biomarkers, cognitive remediation, complex auditory brain-stem response, EEG, psychosis, schizophrenia

## Abstract

Cognitive deficits limit psychosocial functioning in schizophrenia. For many patients, cognitive remediation approaches have yielded encouraging results. Nevertheless, therapeutic response is variable, and outcome studies consistently identify individuals who respond minimally to these interventions. Biomarkers that can assist in identifying patients likely to benefit from particular forms of cognitive remediation are needed. Here, we describe an event-related potential (ERP) biomarker – the auditory brain-stem response (ABR) to complex sounds (cABR) – that appears to be particularly well-suited for predicting response to at least one form of cognitive remediation that targets auditory information processing. Uniquely, the cABR quantifies the fidelity of sound encoded at the level of the brainstem and midbrain. This ERP biomarker has revealed auditory processing abnormalities in various neurodevelopmental disorders, correlates with functioning across several cognitive domains, and appears to be responsive to targeted auditory training. We present preliminary cABR data from 18 schizophrenia patients and propose further investigation of this biomarker for predicting and tracking response to cognitive interventions.

Cognitive impairment is a core feature of schizophrenia that is associated with psychosocial functioning deficits [e.g., Ref. (1, 2)]. Neural network models of cognitive dysfunction in psychosis implicate a distributed neural architecture that includes “higher” cortical regions specialized for integrative cognitive operations as well as neural substrates of lower-level perceptual processing [e.g., Ref. (3, 4)]. Consistent with these models, auditory perceptual deficits have been shown to contribute significantly to impaired cognition (5). Despite the fundamental role of subcortical structures in auditory processing and cognition, neurophysiological characterization of subcortical functioning is largely underrepresented in the schizophrenia literature. The auditory brainstem and midbrain, in particular, include a group of structures that support crucial functions in the representation of auditory information: the cochlear nuclei, superior olivary complex, lateral lemniscus, inferior and superior colliculi, and the auditory thalamus. These structures are sensitive to the subtle cues required for perception of pitch, timing, amplitude and localization of sounds, and they share reciprocal feedback with higher cortical regions through dense ascending and descending pathways (6). This subcortical network has an extremely well-established neurophysiological biomarker of functioning: the ABR. In fact, the ABR is among the most widely used clinical EEG measures with a variety of validated applications including newborn hearing screenings, auditory threshold estimation, intraoperative monitoring of auditory system function, detecting auditory nerve and brain-stem lesions, assessing for the presence of demyelinating conditions, detecting brain death, and determining coma type and recovery prognosis (7).

Despite abundant evidence of auditory system dysfunction in schizophrenia, surprisingly few studies have actually examined ABRs in this population, and results therein have been equivocal. Some studies have found normal ABRs (8, 9) while others have detected abnormal or even missing responses (10–14). Interestingly, abnormal brain-stem activity has been associated with the presence of auditory hallucinations (13, 15), a hallmark symptom experienced by most schizophrenia patients at some point over the course of illness (16). More thorough assessment of brain-stem function in schizophrenia is, therefore, warranted.

One particularly promising subcortical target for future investigation is the inferior colliculus (IC) of the midbrain, a key auditory processing structure and convergence site for auditory pathways ascending from brainstem to cortex (6). The IC is crucial for maintaining fidelity of neural signals generated at the very earliest stages of auditory perception [i.e., in the cochlear nucleus and auditory nerve (17)]. Along with the nearby lateral lemniscus, IC exhibits the ability to phase-lock up to 1,000 Hz concordant with the acoustic properties of relatively complex sinusoidal sounds (18–22). Extant neurophysiologic research on brainstem and midbrain function in schizophrenia has only examined ABRs evoked in response to simple click stimuli (click ABRs), which lack the complexity necessary to evoke this “frequency-following” response. Speech and other more acoustically complex sounds, however, do elicit such a response and could thus provide a more sensitive assessment of brainstem and midbrain neurophysiology in schizophrenia.

## Complex Auditory Brain-Stem Response

**Figure 1:**
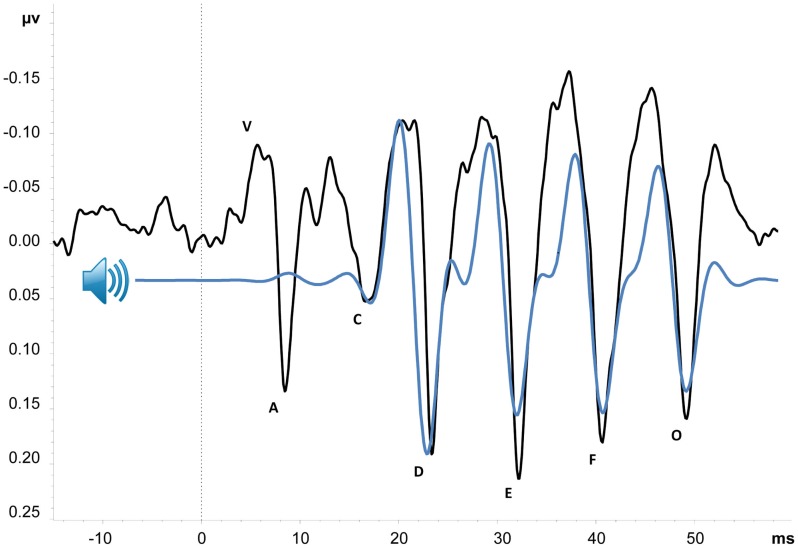
**Grand average cABR from 18 schizophrenia patients**. Black line is average cABR from Cz; peaks are named in accordance with convention [e.g. (27)]. Blue line represents “da” stimulus sound wave, 200 Hz low-pass filtered to better illustrate similarities between cABR and stimulus. Sound wave has also been shifted forward approximately six milliseconds to account for transmission time from cochlea to brainstem. Peaks V–A, C, and O are transient features of the cABR, corresponding with stop consonant onset, consonant-vowel formant transition, and offset of voicing, respectively. Peaks D, E, and F comprise the sustained frequency-following response (FFR) of the vowel sound (Tarasenko et al., in preparation).

The complex auditory brain-stem response (cABR), shown in Figure 1, is an event-related potential (ERP) with an onset approximately 6 ms after presentation of an acoustically complex sound. Its peak amplitudes and latencies correspond with the acoustic properties of its evoking stimulus (23, 24), and it is thought to provide an objective index of the brainstem and midbrain’s representation of complex sounds [cf. Ref. (25)]. Although cABRs can be evoked in response to various types of complex stimuli, here, we primarily focus on those evoked by consonant-vowel speech sounds (e.g., /da/or/mi/). As illustrated in Figure 1, portions of the cABR uniquely correspond to speech stimulus parameters – namely, the stop consonant onset, consonant-to-vowel formant transition, frequency-following response to the vowel sound, and offset of voicing. Neural representation of stimulus pitch, timing, and timber can be derived from the waveform, with high correlation between cABR and stimulus suggesting accurate encoding of sound (25–27). Abnormal cABRs can be characterized by a number of features including small peak amplitudes and long peak latencies relative to the stimulus sound wave (i.e., small stimulus-to-response correlation), low signal-to-noise ratio, weak phase-locking activity, and response variability over time. Such abnormalities have been demonstrated in a number of clinical conditions, including specific language impairment, dyslexia, and autism (25, 28, 29). Older adults have also demonstrated abnormalities in comparison with their younger counterparts (30, 31). Perhaps the most compelling argument for investigating the cABR in schizophrenia comes from Russo et al., who showed that children with autism exhibit abnormal cABRs in the context of intact click-ABRs (29), suggesting that speech and perhaps other complex sounds may offer improved sensitivity over traditional click-ABR measures for detecting brainstem and midbrain dysfunction.

## Utility of the cABR as a Treatment Biomarker in Psychosis

Numerous higher level sequelae of cABR abnormalities have been identified, with robust relationships demonstrated between cABRs and reading abilities (32), phonological processing (33–35), perception of speech in background noise (36), language learning (37), auditory selective attention (38), auditory learning (39), auditory working memory (40), and executive functioning (38). Given that deficits in several of these domains are also commonly present in patients with psychotic illnesses, the cABR may serve as a sensitive index of neural dysfunction occurring within the earliest stages of auditory processing that subsequently cascades “forward” to affect the engagement of higher cortical networks known to underlie cognitive deficits in schizophrenia, or that may otherwise reflect pathology common to both brainstem and higher brain areas.

Among the more intriguing findings from cABR research thus far is that, contrary to conventional beliefs about the “fixedness” of subcortical structures and their relatively passive role in auditory perception (41, 42), brain-stem activity can seemingly be modified as a function of experience with sound. Musicians, presumed to be “auditory experts” due to their extensive training in pitch discrimination and other auditory skills, exhibit stronger correspondence between cABRs and speech stimuli than do non-musicians, and cABR pitch-tracking accuracy is positively correlated with years of musical training (43–45). Language experience can also influence brain-stem activity, as evidenced by greater fidelity of cABRs in tonal language speakers (46) and bilinguals (38). Notably, the benefits of auditory experience on acoustic processing can also be reaped through targeted short-term interventions, with increased cABR fidelity having been found following training programs containing an auditory discrimination training component (47–50). In fact, Skoe et al. (51) demonstrated transient cABR modification after only 15 min of auditory training, leading the authors to characterize the brainstem as a “barometer of rapid auditory learning.”

The malleability of cABRs in response to even a brief course of auditory training is particularly relevant to current treatment development efforts in psychosis. Recently, cognitive training programs have capitalized on existing knowledge of sensory disruption in psychosis by incorporating auditory frequency discrimination exercises that are designed to place implicit and increasing demands on basic auditory perception. Targeted cognitive training (TCT) is one such approach, aiming to improve cognition in part by “sharpening” the fidelity of auditory processing. Data increasingly suggest that targeted “tuning” of underlying neural systems is indeed beneficial for facilitating cognitive recovery in schizophrenia, with patients demonstrating large improvements in auditory-dependent domains of verbal learning and memory and verbal working memory that generalize to enhanced global cognition (*d* = 0.86–0.89) following 50 h of TCT (52). Despite evidence of TCT’s efficacy at the group level, however, individual responses to the training are highly variable, with some patients showing virtually no cognitive improvement even after an extended course of 100 h of training (53). There is, thus, a need to identify ERP biomarkers that are sensitive to neurophysiological changes occurring early in TCT and may predict response to a full “dose” of this and other resource-intensive cognitive interventions.

The identification of biomarkers has been a high priority for psychiatry research, due to mounting evidence of overlapping neural networks that underlie multiple psychiatric illnesses, calling into question the validity of traditional symptom-based diagnostic categories (54, 55). Biomarkers that provide direct assays of the neural circuits underlying clinical phenomena may allow for more precise diagnosis and reliable estimation of benefit from interventions targeting clinically relevant neural circuits (56). Although substantial progress has been made in validating a number of viable candidates (57–62), a “gold standard” ERP biomarker of cognitive dysfunction in psychosis has not yet been established. Scalp ERP measures that currently permeate this literature (e.g., amplitudes and latencies of peaks P50, N1, MMN, P300, etc.) reflect relatively high-level brain responses to sound stimuli and have been shown to sum potentials from multiple cortical source areas (3, 4, 63, 64). Peak cABR measures, in contrast, provide an objective probe of sound representation and have little intra-individual variation in the absence of a systematic auditory training regimen (65); their ability to reliably quantify the degree of disruption present in the auditory signal may thus proffer a unique advantage for accurately diagnosing psychotic disorders and predicting likelihood of benefit from TCT and/or other cognitive, pharmacologic, or combined interventions. Reported adaptations in brain-stem responses early in the course of auditory training [e.g., after only 15 min (51)] could suggest both engagement of the targeted neural system and a capability for neural plasticity required to benefit more broadly from further training.

Importantly, the ERP paradigms used to study cABRs are ideal for inclusion in a biomarker test battery, as they are typically short in duration (approximately 15 min) and require little attention or effort from participants, who typically engage in a distracting visual task during auditory stimulus presentation [see in Ref. (27) for further discussion of task parameters]. Preliminary data, shown in Figure 1, suggest that this type of task is indeed feasible to administer and is well tolerated by schizophrenia patients. A measure of cABR functioning could therefore be easily added to existing batteries to better inform diagnosis and guide subsequent treatment.

## Conclusion

Neurocognitive deficits commonly found in schizophrenia patients may in part reflect dysfunction in lower-level auditory processing mechanisms that have conventionally been studied with click-ABRs and other neurophysiological biomarkers [e.g., Ref. (54, 56, 57, 59, 60, 66, 67)]. The cABR provides an objective, multidimensional measure of sound encoding that is abnormal in some neurodevelopmental disorders, and these deficits are associated with impaired performance across several higher-order cognitive domains. This measure may serve as a sensitive biomarker that predicts or corresponds to therapeutic response to auditory-based cognitive training interventions for schizophrenia.

## Conflict of Interest Statement

Dr. Swerdlow is a consultant for Genco Sciences, Inc. Dr. Light has served as a consultant for Astellas, Forum, and NeuroVerse for matters unrelated to this study. Drs. Tarasenko, Makeig, and Braff report no biomedical financial interests or potential conflicts of interest.
